# Paradoxical Hyperreflexia in a Patient With Classic Guillain-Barré Syndrome

**DOI:** 10.7759/cureus.50184

**Published:** 2023-12-08

**Authors:** Quang Nguyen, Cynthia Janku, Michelle Tan, Rachel Hunsucker, Jeffrey J Li, Tehmina Salahuddin

**Affiliations:** 1 Neurology, California University of Science and Medicine, Colton, USA; 2 Arrowhead Neurosurgical Medical Group, Redlands Community Hospital, Redlands, USA

**Keywords:** clinical electro-diagnostics, lumbar puncture (lp), nerve conduction studies (ncs), guillain-barre syndrome (gbs), guillian-barre syndrome

## Abstract

Background: Guillain-Barré syndrome (GBS) is a rare entity with characteristic features, including progressive ascending paralysis. Patients typically present with progressive symmetrical weakness with areflexia in bilateral lower extremities, which can be confounded by psychiatric comorbidities. This case is unusual in that the patient had paradoxical hyperreflexia and normal CSF protein levels during her initial presentation, later confirmed to be GBS.

Case presentation: Here, we describe the case of a young female with bipolar disorder who presented to the hospital with complaints of week-long bilateral lower leg weakness that started abruptly about a month after an episode of multiple stools of bloody diarrhea. The initial neurological exam revealed 4/5 bilateral lower extremity strength and near global areflexia, excluding a 3+ right patellar reflex, and CSF studies returned normal CSF protein levels.

Based on the clinical presentation of worsening ascending paralysis, electromyography (EMG) findings, and nerve conduction studies (NCS) consistent with an axonal and demyelinating neuropathy, we diagnosed her with the classic form of Guillain-Barré syndrome with paradoxical hyperreflexia.

Imaging results, laboratory findings, treatment decisions, and outcomes of this case are presented.

## Introduction

Guillain-Barré syndrome (GBS) is a rare entity with characteristic features, including progressive ascending paralysis. Patients typically present with progressive symmetrical weakness with hyporeflexia in the bilateral lower extremities. Left untreated, it can continue to ascend and involve upper extremities and, less commonly, respiratory muscle failure. The current incidence of GBS is 0.4 to 1.7 per 100,000 people [1]. The diagnosis is clinical, requiring a proper history and physical exam, particularly if there was a recent gastrointestinal (GI) illness [2]; supporting findings include albuminocytologic dissociation in cerebrospinal fluid (CSF). Electrodiagnostic studies and MR imaging are not typically required in practice but can lend crucial support if atypical clinical features exist. Additionally, psychiatric comorbidities can confound clinical diagnosis, necessitating additional studies [3]. Both factors-atypical clinical features and psychiatric history-were considerations with our patient. Standard of care treatment is either intravenous immunoglobulin (IVIG) or plasma exchange (PLEX) [4], depending on availability, tolerance, and convenience, as both are considered equally efficacious. In our patient’s case, normal CSF protein levels and a complicated psychiatric history were factors that delayed the diagnosis and treatment of GBS. Nerve conduction testing was performed to confirm the diagnosis due to a high degree of clinical suspicion.

## Case presentation

Clinical presentation

A 31-year-old female with a past medical history of bipolar disorder, morbid obesity with a body mass index (BMI) > 40 kg/m2, and cholecystectomy presented to the hospital for a week-long history of abrupt onset progressive bilateral lower extremity weakness. The progressive weakness was accompanied by ascending numbness, consequent falls, and difficulty in ambulation. Pertinent history included a GI illness four weeks prior involving protracted nausea, vomiting, and bloody diarrhea following a hiking trip. The patient had persistent abdominal and lower extremity cramping, nausea, and vomiting, as well as decreased oral tolerance, which caused a 40-lb weight loss over three months. 

Diagnostics and findings

On initial neurologic evaluation, the patient had unremarkable vital signs of a heart rate of 91 beats per minute, a respiratory rate of 18 breaths per minute, a blood pressure of 128/88 mmHg, an oxygen saturation of 99% on room air, and a temperature of 98.2°F. Initial labs revealed hypokalemia and macrocytic anemia (Table 1). 

**Table 1:** Labs on initial neurology encounter CBC: Complete blood count, RBC: Red blood cells, WBC: White blood cells, MCV: Mean corpuscular volume, MCH: Mean corpuscular hemoglobin, RDW: Red cell distribution width, MCHC: Mean corpuscular hemoglobin concentration, BUN: Blood urea nitrogen, CO2: Carbon dioxide, eGFR: estimated glomerular filtration rate

Initial Neuro Encounter Labs (Day 4)		
CBC	Value	Reference Range
Auto WBC	6.5	4.5-11.2 10*3/uL
RBC	2.15	4.00-5.20 10*6/uL
Hemoglobin	8.7	11.5-15.5 g/dL
Hematocrit	25	36%-46%
MCV	117	80.0-100.0 fL
MCH	40.5	26.0-32.0 pg
RDW	16	11.0-15.0%
Platelets	290	120-360 10*3/uL
Platelet Estimate	Adequate	
MCHC	35	33-35 g/dL
Chem Profile		
Sodium	145	135-148 mmol/L
Potassium	3.0	3.5-5.5 mmol/L
Chloride	109	98-110 mmol/L
CO2	25	24-34 mmol/L
BUN	<1.4	8-20 mg/dL
Creatinine	0.59	0.50-1.50 mg/dL
eGFR	123.7	mL/min/1.73m*2
Glucose	122	65-125 mg/dL
Calcium	8.2	8.5-10.5 mg/dL
Phosphorus	2.5	2.4-4.4 mg/dL
Magnesium	2.0	1.6-2.3 mg/dL

**Figure 1:**
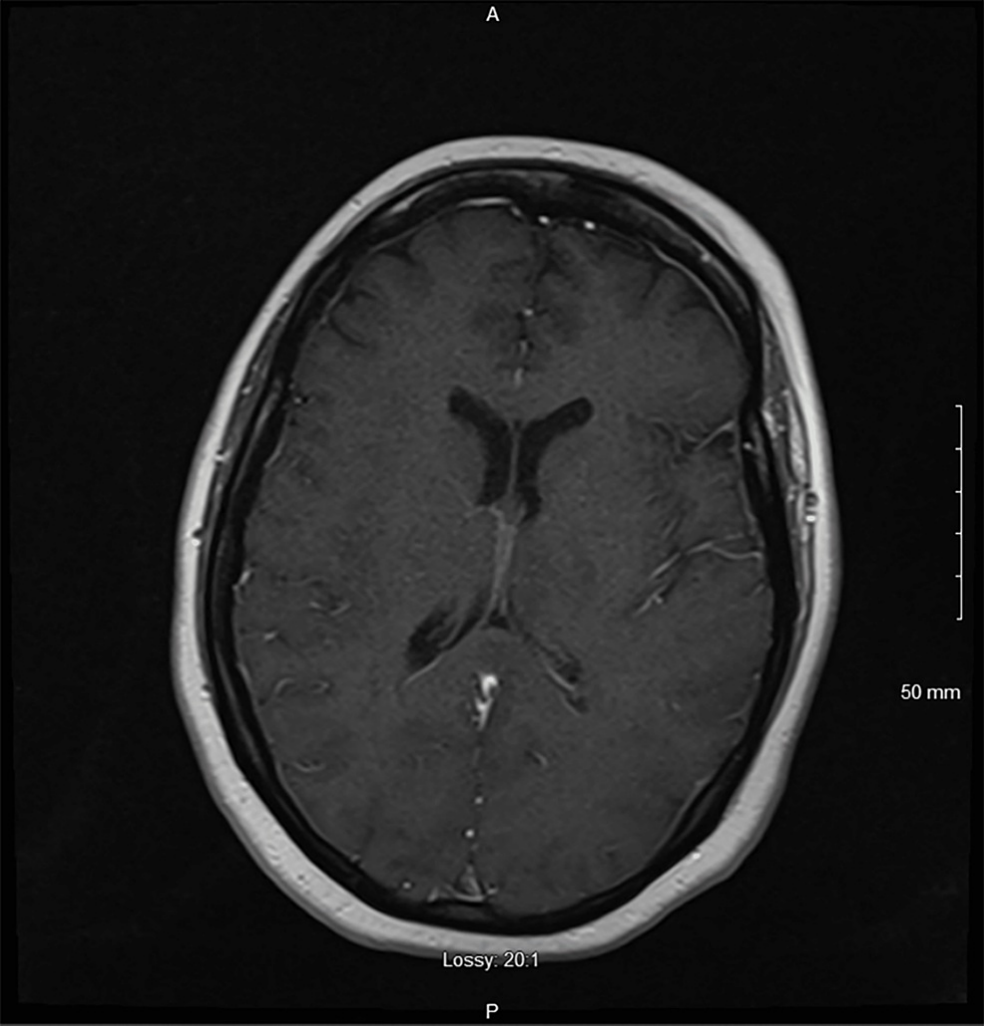
MRI of the brain showed no acute cortical abnormalities.

**Figure 2:**
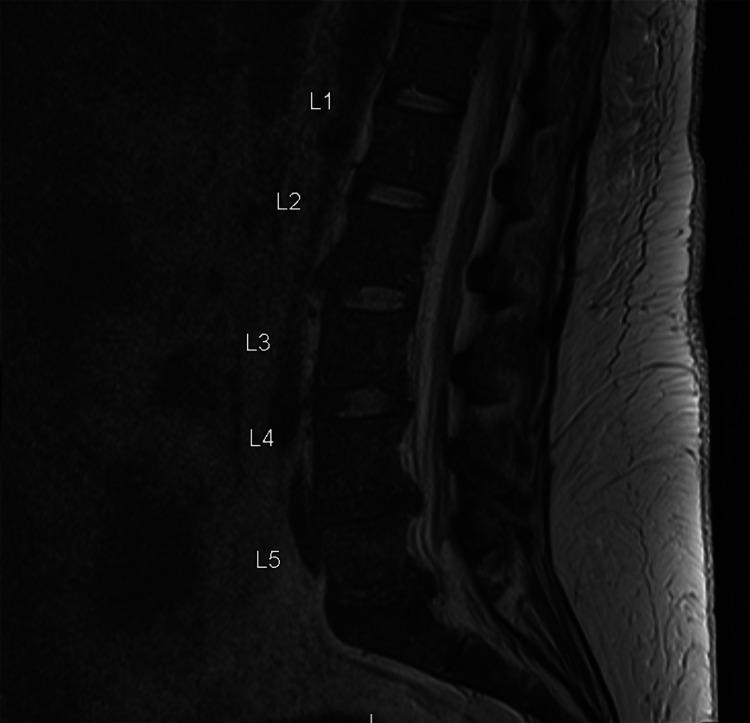
MRI lumbar spine: there are posterior protrusions at the L3-4 through L5-S1 levels. There is no spinal canal stenosis. There is mild to moderate left L5-S1 neural foramen narrowing.

The initial physical exam demonstrated intact cranial nerves, 4/5 bilateral lower extremity muscle strength, and generalized areflexia with a 3+ right patellar reflex. An unusual possible contralateral left bicep to right patellar reflex was also noted, where testing the reflex at the left bicep would induce a reflex-like reaction at the right patella. The patient had 5/5 strength in bilateral dorsiflexion, plantar flexion, shoulder abduction, wrist extension and flexion, and an intact sensation of temperature and proprioception throughout. The patient had intact sensations of temperature, light touch, and proprioception throughout and had right leg drift on examination. To assess the etiology of the patient's weakness, magnetic resonance imaging (MRI) with and without contrast of the neuroaxis was performed. MRI of the brain (Figure 1) and thoracic cord were performed first; MRI of the brain revealed no significant abnormalities, and MRI of the lumbar spine found posterior protrusions at the L3-4 to L5-S1 levels with mild to moderate left L5-S1 neural foraminal narrowing (Figure 2).

**Figure 3:**
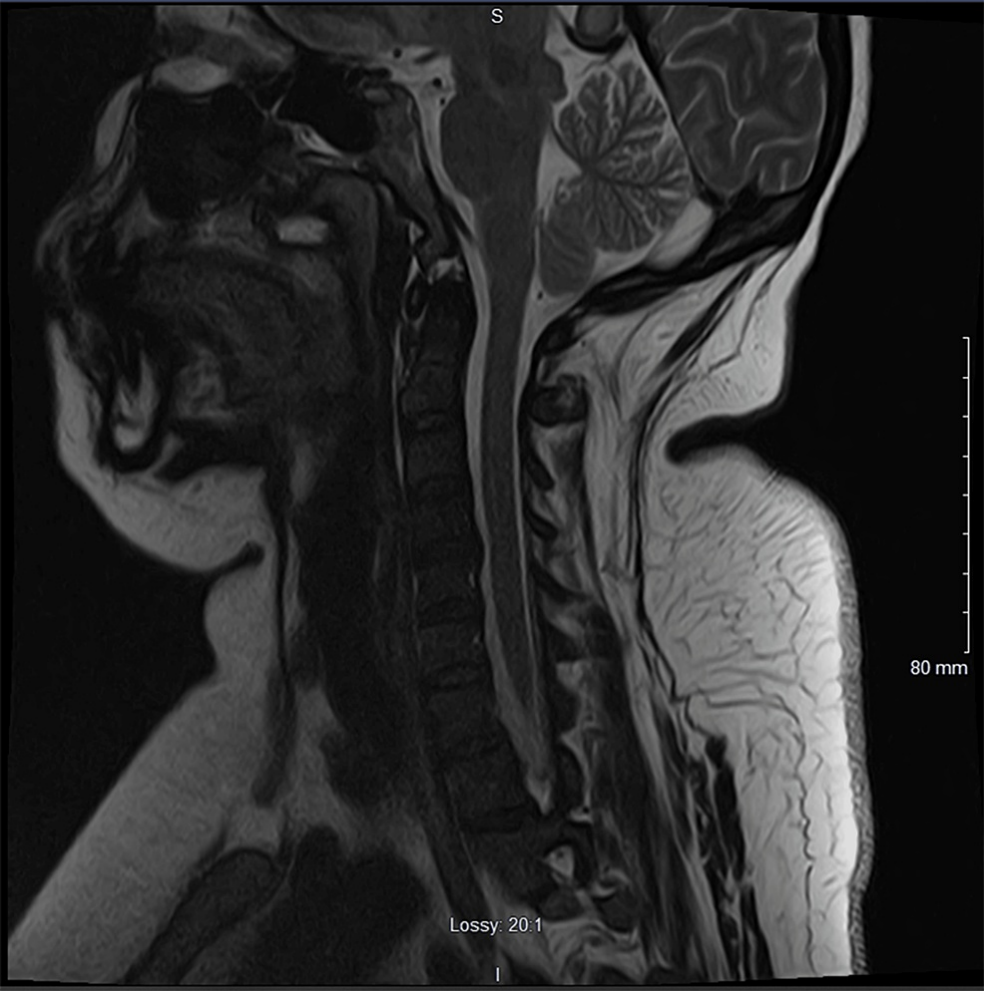
Unremarkable MRI cervical spine.

**Figure 4:**
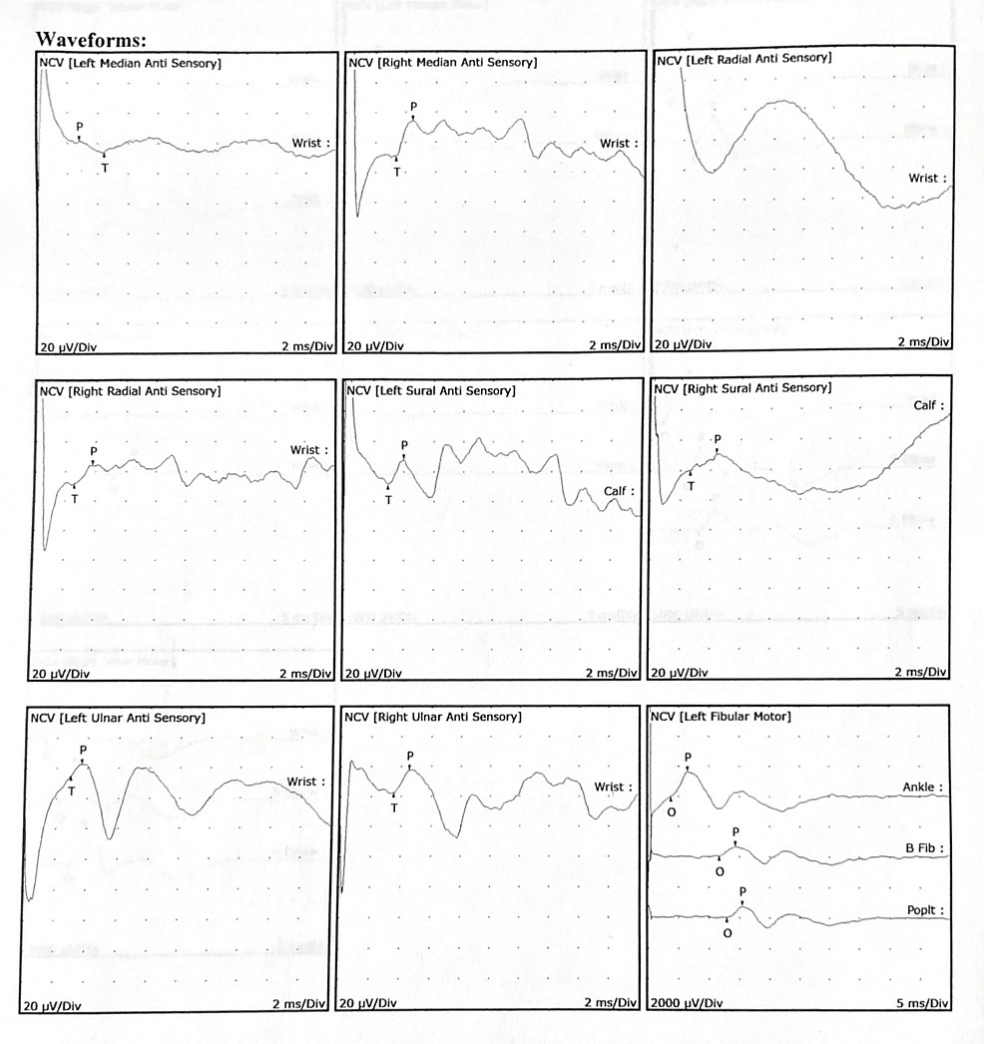
Nerve conduction study waveforms

**Figure 5:**
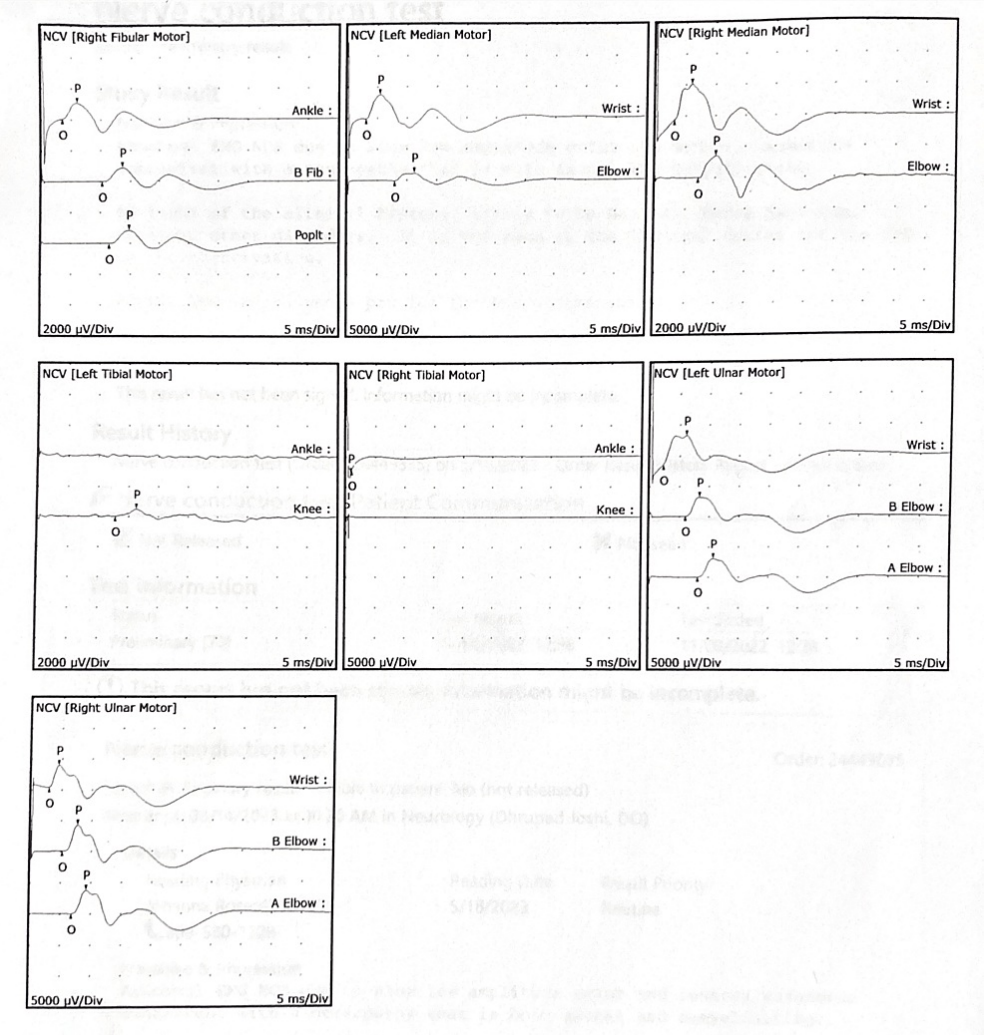
Nerve conduction study waveforms, continued

CSF studies on hospital day two, nine days after the onset of weakness, were unremarkable, showing normal glucose, protein, and cell count. MRI of the cervical spine with and without contrast and nerve conduction studies were also ordered. On hospital day three, the patient exhibited dysmetria and dysdiadochokinesia of the bilateral upper extremities. A cervical spine MRI with and without contrast was performed the following day and revealed no abnormalities (Figure 3). Right patellar hyperreflexia was noted. CSF on hospital day 11 was unchanged with unremarkable findings (Table 2), while the physical exam showed progressing bilateral lower extremity weakness of 2/5 motor strength, 4/5 proximal upper extremity motor strength, and hypoesthesia. At this point, electrodiagnostic studies were conducted and revealed findings consistent with demyelination (Tables 3, 4 and Figures 4, 5). There was a reduced amplitude of the left fibular motor nerve (1.7 mV), right fibular motor nerve (1.0 MV), left median motor nerve (4.0 mV), right median motor nerve (2.3 mV), right tibial motor nerve (0.5 mV), left median sensory nerve (7.2 µV), and left ulnar sensory nerve (7.8 µV). The left tibial motor nerve and the left radial sensory nerve showed no response. There was decreased conduction velocity of the left median motor nerve (Elbow-Wrist 49 m/s), left ulnar motor nerve (Above Elbow-Below Elbow 45 m/s), right median sensory nerve (Wrist- 2nd Digit 31 m/s), right sural sensory nerve (Calf- Lateral Malleolus 32 m/s), and right ulnar sensory nerve (Wrist- 5th Digit 31 m/s). There was prolonged distal peak latency of the right radial sensory nerve (3.8 ms), right sural sensory nerve (4.4 ms), and right ulnar sensory nerve (4.5 ms). With these findings, treatment for GBS was initiated.

**Table 2:** CSF studies on hospital day 12. CSF: Cerebrospinal fluid, VDRL: Venereal disease research laboratory test, IGM: Immunoglobulin M, IGG: Immunoglobulin G

Spinal Fluid	Results	Reference Ranges
Glucose, CSF	76	40-70 mg/dL
Protein, CSF	22	15-45 mg/dL
RBC, CSF	0.0	/mm3
WBC, CSF	0.0	None uL
Appearance, CSF	Clear	Clear
Color, CSF	Colorless	Colorless
Color, Supernatant CSF	Colorless	Colorless
West Nile Virus IGG, CSF	<1.30	IgG <1.30
West Nile Virus IGM, CSF	< .90	IgM
Appearance, Supernatant CSF	Clear	Clear
CSF VDRL	NONREACTIVE	NONREACTIVE

**Table 3:** Nerve conduction studies summary table

Nerve Conduction Studies											
Anti Sensory Summary Table											
Stim Site	NR	Peak (ms)	Norm Peak (ms)	P-T Amp (µV)	Norm P-T Amp	Site 1	Site 2	Delta-P (ms)	Dist (cm)	Vel (m/s)	Norm Vel (m/s)
Left Median Anti Sensory (2nd Digit)											
Wrist		2.8	<3.6	7.2	>10	Wrist	2nd Digit	2.8	14.0	50	>39
Right Median Anti Sensory (2nd Digit)											
Wrist		4.5	<3.6	24.0	>10	Wrist	2nd Digit	4.5	14.0	31	>39
Left Radial Anti Sensory (Base 1st Digit)											
Wrist	NR		<3.1			Wrist	Base 1st Digit		0.0		
Right Radial Anti Sensory (Base 1st Digit)											
Wrist		3.8	<3.1	12.4		Wrist	Base 1st Digit	3.8	0.0		
Left Sural Anti Sensory (Lat Mall)											
Calf		4.0	<4.0	16.7	>5.0	Calf	Lat Mall	4	14.0	35	>35
Right Sural Anti Sensory (Lat Mall)											
Calf		4.4	<4.0	11.1	>5.0	Calf	Lat Mall	4.4	14.0	32	>35
Left Ulnar Anti Sensory (5th Digit)											
Wrist		3.6	<3.7	7.8	>15.0	Wrist	5th Digit	3.6	14.0	39	>38
Right Ulnar Anti Sensory (5th Digit)											
Wrist		4.5	<3.7	15.3	>15.0	Wrist	5th Digit	4.5	14.0	31	>38
Motor Summary Table											
Stim Site	NR	Peak (ms)	Norm Peak (ms)	P-T Amp (µV)	Norm P-T Amp	Site 1	Site 2	Delta-P (ms)	Dist (cm)	Vel (m/s)	Norm Vel (m/s)
Left Fibular Motor (Ext Dig Brev)											
Ankle		3.8	<6.1	1.7	>2.5	B Fib	Ankle	7.9	29.0	37	>38
B Fib		11.7		0.6		Poplt	B Fib	1.3	10.0	77	>40
Poplt		13.0		0.7							
Right Fibular Motor (Ext Dig Brev)											
Ankle		3.5	<6.1	1.0	>2.5	B Fib	Ankle	6.8	30.0	44	>38
B Fib		10.3		0.9		Poplt	B Fib	1.2	10.0	83	>40
Poplt		11.5		0.8							
Left Median Motor (Abd Poll Brev)											
Wrist		3.1	<4.2	4.0	>5	Elbow	Wrist	4.9	24.0	49	>50
Elbow		8.0		1.2							
Right Median Motor (Abd Poll Brev)											
Wrist		3.1	<4.2	2.3	>5	Elbow	Wrist	4.0	21.0	53	>50
Elbow		7.1		1.4							
Left Tibial Motor (Abd Hall Brev)											
Ankle	NR		<6.1		>3.0	Knee	Ankle		0.0		>35
Knee		12.8		0.5							
Right Tibial Motor (Abd Hall Brev)											
Ankle		0.9	<6.1	0.5	>3.0	Knee	Ankle		0.0		>35
Knee				0.7							
Left Ulnar Motor (Abd Dig Min)											
Wrist		2.3	<4.2	4.7	>3	B Elbow	Wrist	3.7	25.0	68	>53
B Elbow		6.0		3.1		A Elbow	B Elbow	2.2	10.0	45	>53
A Elbow		8.2		3.0							
Right Ulnar Motor											
Wrist		2.9	<4.2	3.6	>3	B Elbow	Wrist	2.3	21.0	91	>53
B Elbow		5.2		4.2		A Elbow	B Elbow	1.7	10.0	59	>53
A Elbow		6.9		3.7							

**Table 4:** Nerve conduction studies left/right comparison

Nerve Conduction Studies											
Anti Sensory Left/Right Comparison											
Stim Site	L Lat (ms)	R Lat (ms)	L-R Lat (ms)	L Amp (µV)	R Amp (µV)	L-R Amp (%)	Site 1	Site 2	L Vel (m/s)	R Vel (m/s)	L-R Vel (m/s)
Median Anti Sensory (2nd Digit)											
Wrist	2.8	4.5	1.7	7.2	24.0	70.0	Wrist	2nd Digit	50	31	19
Radial Anti Sensory (Base 1st Digit)											
Wrist		3.8			12.4		Wrist	Base 1st Digit			
Sural Anti Sensory (Lat Mall)											
Calf	4.0	4.4	0.4	16.7	11.1	33.5	Calf	Lat Mall	35	32	3
Ulnar Anti Sensory (5th Digit)											
Wrist	3.6	4.5	0.9	7.8	15.3	49.0	Wrist	5th Digit	39	31	8
Motor Left/Right Comparison											
Stim Site	L Lat (ms)	R Lat (ms)	L-R Lat (ms)	L Amp (µV)	R Amp (µV)	L-R Amp (%)	Site 1	Site 2	L Vel (m/s)	R Vel (m/s)	L-R Vel (m/s)
Fibular Motor (Ext Dig Brev)											
Ankle	3.8	3.5	0.3	1.7	1.0	41.2	B Fib	Ankle	37	44	7
B Fib	11.7	10.3	1.4	0.6	0.9	33.3	Poplt	B Fib	77	83	6
Poplt	13.0	11.5	1.5	0.7	0.8	12.5					
Median Motor (Abd Poll Brev)											
Wrist	3.1	3.1	0.0	4.0	2.3	42.5	Elbow	Wrist	49	53	4
Elbow	8.0	7.1	0.9	1.2	1.4	14.3					
Tibial Motor (Abd Hall Brev)											
Ankle		0.9			0.5		Knee	Ankle			
Knee	12.8		12.8	0.5	0.7	28.6					
Ulnar Motor (Abd Dig Min)											
Wrist	2.3	2.9	0.6	4.7	3.6	23.4	B Elbow	Wrist	68	91	23
B Elbow	6.0	5.2	0.8	3.1	4.2	26.2	A Elbow	B Elbow	45	59	14
A Elbow	8.2	6.9	1.3	3.0	3.7	18.9					

Treatment and outcome

The patient was treated with IVIG 0.4mg/kg/day for five days with simultaneous physical therapy during admission and a plan for acute rehab on discharge. At discharge, the patient reported subjective strength improvement, which correlated with objective physical exam findings.

Before treatment, the patient had 2/5 bilateral lower extremity strength and 3/5 bilateral upper extremity strength with diffuse hyporeflexia and intact sensation of temperature and proprioception throughout. After five days of IVIG treatment, there was improved 3/5 right lower extremity strength and 4/5 left lower extremity strength, but persisting diffuse hyporeflexia.

## Discussion

The diagnosis of Guillain-Barré syndrome (GBS) is primarily a clinical diagnosis that can be supported by objective findings. GBS is a relatively rare post-infectious entity that typically presents with symmetric ascending weakness and is widely associated with gastrointestinal infections, such as *Campylobacter jejuni*, although other infections have been reported. Upper respiratory infections are another common cause of GBS [5], and the influenza vaccine is also shown to be a cause of reactivation of GBS [6]. Another case report reflected that aspiration pneumonia from drug overdose was another possible cause leading to the development of GBS [7]. 

Typical clinical features of classical GBS include rapidly progressive bilateral ascending weakness with decreased or absent reflexes, distal paresthesias, or sensory loss. GBS, however, includes variants with atypical presentations, such as pure motor weakness without sensory loss or symptoms isolated to just the upper or lower limbs [2]. 

An interesting association is that a prior history of autoimmune disease, Crohn’s disease, or GBS, is associated with a higher risk of developing bipolar disorder [8]. Another study described patients with bipolar disease as having increased antibodies in their CSF, reflecting inflammation [9]. It’s been discussed in other papers that severe neurological emergencies, such as GBS, can be misdiagnosed due to psychiatric illness disorders [10]. Our patient’s extensive history of significant weight loss and bloody diarrhea with differing timelines initially pointed at a functional neurologic disorder, as the CSF studies were not consistent with GBS. GBS has been misdiagnosed as conversion disorder [11]; therefore, the reliance of the CSF is important to consider in diagnosis. 

Atypical presentations and comorbid psychiatric disease often lead to delays in diagnosis and the risk of progression of symptoms; supportive objective findings, such as cerebrospinal fluid (CSF) studies, can help confirm the diagnosis in patients with suspicious histories and physical exam findings. Typically in GBS, we would expect to see increased protein in the CSF studies, but this was not the case for our patient.

In our case report, the combined presence of normal CSF and atypical hyperreflexia guided our initial diagnosis away from GBS. A systematic review reflected that there were 44 case reports of GBS with hyperreflexia, and 56% of those patients had antecedent diarrhea [12], similar to our patient. A review done in Japan showed that patients with suspected GBS but had hyperreflexia were able to be diagnosed with GBS through electrodiagnostic studies [13]. The patients described in this study were classified as having AMAN or AIDP [13]. 

Similarly, bilaterally decreased conduction velocities on the EMG and NCS proved to be diagnostic for GBS in our patient. Though we did not estimate the degree of temporal dispersion and conduction block, we were confident enough in the clinical picture to initiate treatment. The pathogen responsible for our patient’s protracted GI illness was unidentified, but her presentation, excluding the hyperreflexia, was typical for GBS, and strong clinical suspicion drove further investigation. 

Given that GBS is associated with a risk of upper extremity involvement and, less commonly, respiratory muscle failure, advanced diagnostics can be an important tool for confirming GBS in patients with strong clinical suspicion when CSF is inconclusive. 

## Conclusions

This case demonstrates the utility of advanced diagnostics when encountering atypical or confounding factors and results, such as the patient's hyperreflexia and normal CSF protein, in a patient with a history and presentation suspicious for GBS. The results of further investigation with advanced diagnostics enabled us to initiate prompt treatment and produced a good patient outcome.
